# ERK1/2-EGR1-SRSF10 Axis Mediated Alternative Splicing Plays a Critical Role in Head and Neck Cancer

**DOI:** 10.3389/fcell.2021.713661

**Published:** 2021-09-20

**Authors:** Sandhya Yadav, Deepak Pant, Atul Samaiya, Neetu Kalra, Sanjay Gupta, Sanjeev Shukla

**Affiliations:** ^1^Department of Biological Sciences, Indian Institute of Science Education and Research Bhopal, Bhopal, India; ^2^Bansal Hospital, Bhopal, India; ^3^VIT Bhopal, Bhopal, India; ^4^Cancer Research Institute, Advanced Centre for Treatment, Research and Education in Cancer, Tata Memorial Centre, Navi Mumbai, India; ^5^Homi Bhabha National Institute, Training School Complex, Mumbai, India

**Keywords:** alternative splicing, ERK/MAPK, HNC (Head and Neck Cancer), SRSF10 (Serine And Arginine Rich Splicing Factor 10), Egr1 (early growth response protein 1)

## Abstract

Aberrant alternative splicing is recognized to promote cancer pathogenesis, but the underlying mechanism is yet to be clear. Here, in this study, we report the frequent upregulation of SRSF10 (serine and arginine-rich splicing factor 10), a member of an expanded family of SR splicing factors, in the head and neck cancer (HNC) patients sample in comparison to paired normal tissues. We observed that SRSF10 plays a crucial role in HNC tumorigenesis by affecting the pro-death, pro-survical splice variants of BCL2L1 (BCL2 Like 1: BCLx: Apoptosis Regulator) and the two splice variants of PKM (Pyruvate kinase M), PKM1 normal isoform to PKM2 cancer-specific isoform. SRSF10 is a unique splicing factor with a similar domain organization to that of SR proteins but functions differently as it acts as a sequence-specific splicing activator in its phosphorylated form. Although a body of research studied the role of SRSF10 in the splicing process, the regulatory mechanisms underlying SRSF10 upregulation in the tumor are not very clear. In this study, we aim to dissect the pathway that regulates the SRSF10 upregulation in HNC. Our results uncover the role of transcription factor EGR1 (Early Growth Response1) in elevating the SRSF10 expression; EGR1 binds to the promoter of SRSF10 and promotes TET1 binding leading to the CpG demethylation (hydroxymethylation) in the adjacent position of the EGR1 binding motif, which thereby instigate SRSF10 expression in HNC. Interestingly we also observed that the EGR1 level is in the sink with the ERK1/2 pathway, and therefore, inhibition of the ERK1/2 pathway leads to the decreased EGR1 and SRSF10 expression level. Together, this is the first report to the best of our knowledge where we characterize the ERK 1/2-EGR1-SRSF10 axis regulating the cancer-specific splicing, which plays a critical role in HNC and could be a therapeutic target for better management of HNC patients.

## Introduction

Head and neck cancer (HNC) is a heterogeneous disease that includes a variety of tumors that originate from gingivobuccal complex (buccal mucosa, alveolus, retromolar trigone, and gingiva), tongue, lip, palate, and floor of the mouth (Nigro et al., 2017). According to the world health organization 2018 data, cancer is the second leading cause of death globally, accounting for one in six deaths. HNC is the sixth most common cancer worldwide (Kumar, 2017), with an incidence rate of 650,000 new cases every year (Kumar, 2017) and more than 350,000 deaths every year (Parkin et al., 2005). HNC arise from the mucosa of the oral cavity and are epithelial in origin, therefore, classified as squamous cell carcinomas. Mainly, HNC patients are detected at a late stage and thus are associated with poor survival rates (Garg and Karjodkar, 2012). An early clinical diagnosis of HNC may improve the survival rate with advancement in therapeutic options. Therefore, it is of great importance to identify new molecular targets for HNC treatment.

Almost all human genes undergo alternative splicing (AS), a highly regulated process, and studies evident that any deregulation in the AS process contributes to tumor progression (Srebrow and Kornblihtt, 2006), including HNC (Yadav et al., 2019). Cancer cells exhibit a remarkable alteration in the splicing process and generate specific splicing isoforms that not only act as drivers of cancer progression but contribute significantly to cancer hallmarks (Ladomery, 2013). The expression of cancer-specific isoform is very tightly regulated by RNA splicing regulators, which recently emerged as a new class of oncoproteins (Dvinge et al., 2016). The RNA splicing regulators involve the group of proteins called splicing factors that include mainly two groups of proteins SR (serine and arginine-rich proteins) family and HnRNP (heteronuclear ribonuclear proteins) family members. These splicing regulators bind to the specific sequence of the target genes and affect inclusion or exclusion of the exon, thereby regulates the expression of different transcripts from a single gene which further controls the key cellular process. A large number of splicing factors have been reported to be deregulated in multiple cancer types (Boukakis et al., 2010; Silipo et al., 2015) and thus found to be responsible for aberrant AS (Gupta et al., 2020). The deregulation of these splicing factors is governed by the triggering of several cellular signaling pathways, eventually leading to altered regulation of AS events (Blaustein et al., 2007). This highlights the importance of continued elucidation of the key signaling pathways, which contributes to the process of carcinogenesis.

Serine and Arginine Rich Splicing Factor 10 (SRSF10) is an atypical member of the SR protein family with a domain organization similar to SR proteins. SRSF10 is characterized recently (Cowper et al., 2001), and has been shown to act as a splicing repressor when activated by dephosphorylation (Shin et al., 2004). Later, the subsequent study reported that the phosphorylated form of SRSF10 could function as a splicing activator in a sequence-dependent manner. In continuation with these reports, SRSF10 was shown to play an important role in the AS process by regulating the exon inclusion both positively and negatively, which depends on its binding at pre mRNA relative to an alternative exon. The key role of SRSF10 is studied in the developmental process using several model systems (Li et al., 2014; Wei et al., 2015). It has been reported to affect colorectal cancer progression by modulating the AS of several genes (Zhou et al., 2014b). These studies strongly suggest that splicing factors play a peculiar role in specific tissue or cells. The function of SRSF10 overexpression in HNC has remained unexplored as well as the underlying molecular pathway that regulates the expression of SRSF10 has not yet been studied to the best of our knowledge.

Here in this study, we strongly highlight the tumorigenic role of SRSF10 in HNC. Importantly, we explored the key pathway for the over-expression of SRSF10 for the first time. Interestingly, we observed that inhibiting ERK signaling pathway results in the downregulation of SRSF10 by affecting the EGR1 expression level. Our data demonstrate that EGR1 regulates the expression level of SRSF10 by recruiting TET1 at the demethylated EGR1 binding site. Conclusively, we have dissected the ERK-EGR1-SRSF10 axis, which plays a critical role in HNC by directing the splicing of tumor-specific isoforms and could be a potential target for better management of HNC patients.

## Materials and Methods

### Cell Culture

The two mammalian HNC cell lines were used in this study; BICR10 (Buccal mucosa squamous carcinoma) and H157 (human oral squamous cell carcinoma) were obtained from the European Collection of Authenticated Cell Culture (ECACC; Salisbury, United Kingdom) in May 2014. These two head and neck cell lines BICR10 (ECACC 04072103) and H157 (ECACC 07030901), were cultured in the ECACC recommended media, supplemented with the 10% Fetal Bovine Serum (Sigma, F7524), 2 mmol L-glutamine (Invitrogen, 25030081) and 100 units/ml of penicillin and streptomycin, and 0.5 μg/ml sodium hydrocortisone succinate at 37°C with 5% CO_2_.

### Head and Neck Cancer Sample Collection

Informed consent was obtained from head and neck patients undergoing surgery at Bansal Hospital, Bhopal, India. After surgery, tumor tissues with paired adjacent normal tissues were collected and snap-frozen immediately and stored at −80°C until use. One pair of tumor tissues and adjacent normal tissues were collected in RNAlater (Sigma, R0901) separately. The Institute Ethics Committee approved this study, and the clinical characteristics of patients used in this are presented in Supplementary Table 2.

### Microarray Data Analysis

Gene expression profiles analyzed in this study were collected from Gene Expression Omnibus GEO (Barrett et al., 2013). Microarray platform with specific probes was mapped to the gene symbols with appropriate annotation files. The expression values of genes with more than one probe were averaged using DNA Chip Analyzer software and considered for the analysis. SRSF10 gene expression values were extracted from normalized and log2 transformed oral tumor profiles. The significant difference in the gene expression between normal and oral tumor was then calculated using the student’s test (two tailed). *p*-value less than 0.05 was considered significant. GraphPad Prism was used to generate the box plots.

### Oncomine Data Analysis

The expression of SRSF10 was examined in Oncomine (Rhodes et al., 2004), HNC profiles were selected for further investigation. The analyzed expression data and graphs were exported for representation.

### Survival Data

Disease-free survival analysis was done using The Cancer Genome Atlas (TCGA) dataset on the GEPIA 2.0 online platform (Gene Expression Profiling Interactive Analysis). A survival curve was generated for patients across all HNSC subtypes using disease-free survival analysis. The patients were divided into high and low SRSF10 cohorts by keeping 30 and 70% as the cutoff value, respectively.

Overall survival analysis was performed by extracting clinical information of HNC patients from the GSE26549 dataset. The samples were divided into SRSF10_high and SRSF10_low group based on the mean of SRSF10 expression across all samples. Survival curve analysis was then performed using Log Rank test in GraphPad Prism (La Jolla, CA, United States).

### RNA Interference

The BICR10 and H157 HNC cells were infected with the lentivirus containing small hairpin RNA (shRNA) purchased from Sigma (Saint Louis, United States) and specific to SRSF10 (sh SRSF10) and eGFP (sh control) using 8 μg/ml polybrene containing media. HNC cells were selected with 0.8 μg/ml puromycin for 2 days. Post selection with puromycin cells was used for further experiments.

### Oligo Sequence of shRNAs

**Table:** 

**shRNA**	**Sequence**
sheGFP	5′-CCGGTACAACAGCCACAACGTCTATCTCGAGATAGAC GTTGTGGCTGTTGTATTTTT-3′
shSRSF10_1	5′-CCGGGCCGAAGTTATGAAAGGAGGACTCGAGTCCTC CTTTCATAACTTCGGCTTTTTG-3′
shSRSF10_2	5′-CCGGCGGCGTGAATTTGGTCGTTATCTCGAGATAAC GACCAAATTCACGCCGTTTTTG-3′
shEGR1_1	5′-CCGGCATCTCTCTGAACAACGAGAACTCGAGTTCTC GTTGTTCAGAGAGATGTTTT-3′
shEGR1_2	5′-CCGGCTGTCTACTATTAAGGCCTTTCTCGAGAAAGG CCTTAATAGTAGACAGTTTTT-3′

### Genomic DNA Isolation

Genomic DNA was isolated from BICR10 cells using mammalian genomic DNA isolation kit (Sigma, G1N70) and according to the manufacturer’s instruction.

### Cloning

The fragments of SRSF10 promoter region were generated by PCR using human genomic DNA as a template and subcloned into the *Nhe*I and *Hin*dIII sites of pGL3-Basic vector. The human genomic DNA was isolated from BICR10 cell lines. SRSF10 promoter fragments from −1,153 to +333, −922 to +333, −333 to +333, −200 to +333, −100 to +333, and +30 to +333 were amplified by using the primers as listed in Supplementary Table 1. PCR conditions is as follows: pre-degeneration for 3 min at 95°C, denaturation for 30 s at 95°C, annealing at 58°C for 30 s and extension at 72°C for 7 min. PCR reactions were carried out for 35 cycles, and PCR products were visualized in 1% agarose gels containing ethidium bromide under UV transillumination.

The PCR product and PGL3-Basic vehicle plasmid were digested with restriction enzyme *Hin*dIII (Takara bio science, 1615) and *Nhe*I (Takara bio science, 1622, 1241A) at 37°C for 2 h. The fragment of PCR product and PGL3-Basic vehicle plasmid was mixed with 1 μl T4 ligase buffer and 1 μl DNA ligase (New England Biolabs, M0202S) and added water to make up the volume up to 20 μl incubated at 22°C for 1 h and then transformed into *E. Coli*. The pGL3-SRSF10 promoter containing vector plasmid was extracted, and all constructs were verified by DNA sequencing.

The EGR1 overexpression plasmid was constructed by amplifying a 1.632 kb EGR1 fragment from BICR10 cDNA using Phusion high fidelity DNA polymerase (NEB, M053) and EGR1 primers (Supplementary Table 1). The product was cloned between the sites *Eco*RI (Takara bio science, 1611) and *Xho*I (Takara bio science, 1635) pFLAG-CMV 1a expression vector (Agilent Technologies, Santa Clara, CA, United States).

### Luciferase Assay

BICR10 cells (5 × 10^4^) were seeded in a 24-well plate and incubated in CO_2_ incubator for 12 h. The cells were transfected with different SRSF10 promoter-luciferase construct as well as pRL-TK Renilla luciferase plasmids and again incubated in CO_2_ incubator for 48 h. The cells were lysed with freshly prepared (150 μl/well) of passive lysis buffer. Transferred the 50 μl of each lysate into the wells of a white 96-well assay plate. Add 50 μl of luciferase lysis buffer per well and then incubated at room temperature (RT) for 2 min with shaking. The firefly luciferase activity was measured in a GloMax Multi Detection System (Promega). The relative luciferase activity can be determined by dividing the firefly luciferase activity with the Renilla luciferase activity. The relative values are represented as mean ± SD of triplicate values from a representative experiment.

### Cell Viability/MTT Assay

BICR10 and H157 cells (2 × 10^6^) were seeded in six-well cell culture plates. After 24 h, sh_SRSF10 and sh_control was transfected, and then selected the transfected cells with puromycin. After selecting these transfected cells with puromycin, the cells were seeded in 96-well culture plated (3 × 10^3^/well) for 12, 24, 36, 48, and 72 h (in triplicate for each condition). The 20 μl of MTT (Sigma, M2128) stock solution (2 mg/ml) was added to each well in addition with 100 μl media and incubated in a CO_2_ incubator for at least 2 h. After incubation, the formazan crystals formed from MTT tetrazolium salt were solubilized using dimethyl sulfoxide. The numbers of viable cells were calculated by measuring the optical density using plate reader BioTek Eon (BioTek, Winooski, United States).

### Wound Healing Assay

After selecting these transfected cells with puromycin, the cells were seeded in a 12-well plate, and upon reaching 100% confluence, the wound was created using a sterile 200 μl pipette tip and washed with 1XPBS two times to remove cell debris. The wounded area was marked in each well on the bottom of plates, and images were captured at 0, 12, 24 h with an inverted microscope. The wound width was measured using Image J software.

### Invasion Assay

After the puromycin selection, 2 × 10^4^ cells were then added to an upper chamber of a transwell (Corning, NY, United States) above a Matrigel layer (Corning, Bedford, MA, United States) and incubated for the next 24 h in a CO_2_ incubator. The cells migrated to the lower chamber of transwell were then fixed in 4% paraformaldehyde solution and then stained with 0.05% crystal violet solution, and images were taken using an inverted microscope (Olympus, Tokyo, Japan).

### Colony-Forming Assay

After puromycin selection, 1 × 10^3^ cells were seeded in the fresh six-well cell culture plate and were maintained in 0.5 μg/ml puromycin-containing media for 12 days. Cells were then fixed using methanol and acetic acid (3:1) for 5 min and then washed with 1× PBS. Cells were then stained with 0.05% crystal violet for 30 min. Then, cells were washed with 1× PBS, and plates were dried for 30 min at room temperature and scanned. Colonies were counted using ImageJ software (La Jolla, CA, United States).

### Immunoblotting

Cells were pelleted and lysed with urea lysis buffer (8M Urea: Sigma IU5378, 2M Thiourea: Sigma 11149, 10% CHAPS: Sigma C9426, 10% Dithiothreitol: Sigma D9779) and kept at 4°C for 30 min. After incubation, the lysate was centrifuged at 14,000 rpm for 2 h, and the supernatant was collected in a 1.5 ml microcentrifuge tube and stored at –80°C. The proteins were then separated by sodium dodecyl sulfate-polyacrylamide gel electrophoresis and transferred to polyvinylidene difluoride (PVDF) membrane (Millipore). The protein-containing PVDF membrane was blocked with 5% non-fat milk in tris buffered saline with 0.05% Tween 20 (TBST) for 30 min. The membrane were then probed with following primary antibodies: anti-SRSF10 (Sigma, HPA053831), anti-pERK (CST, 9101S), anti-ERK (CST, 9102S), anti-EGR1 (CST, 4154S), anti-flag (Novus Biologicals, NBP1-06712SS), anti-PKM2 (CST 4053S), anti-PKM1 (CST 7067S), and anti-GAPDH (CST 5174S). Anti-GAPDH was used as loading controls for protein assays. After 2 h of incubation with primary antibody at RT, membranes were then washed with 1× TBST then again incubated with secondary antibodies for 45 min at RT. The probed PVDF membranes were washed with TBST, and the bands were visualized using an odyssey membrane scanning system (Li-cor Biosciences, Bad Homburg, Germany).

### Hydroxymethylation Dependent Immune Precipitation

Genomic DNA was isolated from BCR10 cells using genomic DNA isolation kit, and hydroxymethylation dependent immune precipitation (hMeDIP) assay were performed as per the protocol previously (Singh et al., 2017). Briefly, genomic DNA was first sonicated, and 3 μg of the sonicated DNA was incubated overnight at 4°C with 5-hydroxy-methyl cytosine antibody (Sigma, MABE251) and normal mouse IgG antibody (Calbiochem, NI03). 5% input and immunoprecipitated fractions were analyzed by qRT-PCR in duplicate using the SYBR Green master mix (Promega, A6002) and specific primers (Supplementary Table 1) across the promoter regions. Normalization was performed with input. Resultant values were then normalized relative to the mouse Ig control IP values for the primer set, and the student’s *t*-test was used to identify the significance between two different groups. *p* < 0.05 was considered statistically significant.

### Chromatin Immune Precipitation

Chromatin immune precipitation (ChIP) assay was performed as described previously (Singh et al., 2017). Briefly, the chromatin was sonicated, and about 25 μg of chromatin was immunoprecipitated using antibody of interest, followed by overnight incubation at 4°C. The following antibodies were used for ChIP: Anti-EGR1 (CST, 4154S), Anti TET1 (Novus, 1462), and Normal rabbit IgG (CST, 2729S). Immunoprecipitated fractions and 5% input were analyzed by quantitative real-time PCR in duplicate using the SYBR Green Master mix (Promega, A6002) and specific primers (Supplementary Table 1) across the promoter regions.

### Lactate Assay

The BICR10 cells (3 × 10^5^) were infected with lentivirus containing shRNA specific for SRSF10 gene in 6 well cell culture plates, and after 4 days, lactate assay was performed according to the manufacturers instruction. Briefly, an equal number of cells were homogenized in the presence of lactate assay buffer provided in lactate assay kit (Sigma, MAK064) and centrifuged at 13,000 *g* for 10 min. Lactate assay was then performed in 96-well plate, and lactate levels were measured with a plate reader at an optical density of 570 nm.

### Glucose Uptake Assay

The BICR10 cells (3 × 10^5^) were infected with lentivirus containing shRNA specific for SRSF10 gene in 6 well cell culture plates, and after 4 days glucose assay was performed according to the manufacturers instruction. Briefly, an equal number of cells were homogenized in the presence of glucose assay buffer provided in glucose uptake assay kit (Sigma, MAK083) and centrifuged at 13,000 *g* for 10 min. Glucose assay was then performed in a 96-well plate, and glucose levels was measured with a plate reader at an optical density of 570 nm.

### Caspase 3/7 Assay

The BICR10 cells (3 × 10^5^) were infected with lentivirus containing shRNA specific for SRSF10 gene in 6 well cell culture plates, and after 4 days, caspase activity was measured. In another set of experiment, post puromycin selection, 4 days later the cells were treated with 30 μM concentration of z-VAD-FMK pan-caspase inhibitor (Sigma, V116) after 24 h the caspase activity was measured. The caspase 3/7 activation was measured using the caspase 3/7 assay kit (Sigma, CASP3F) recommended by the manufacturer. Luminescence readings were taken using a Glomaz multi detection system (Promega).

### Statical Analysis

Statistical analyses were performed using the GraphPad Prism5 (La Jolla, CA, United States). In the bar graph, Student’s *t*-test was used to compare the differences between the two groups. The differences were considered statistically significant with the ^∗^*p* < 0.05, ^∗∗^*p* < 0.01, ^∗∗∗^*p* < 0.001, and non-significant difference (*p* > 0.05).

## Results

### The Upregulation of the Splicing Factor SRSF10 in HNC Patient Samples Is Inversely Correlated With HNC Patient Survival

**FIGURE 1:**
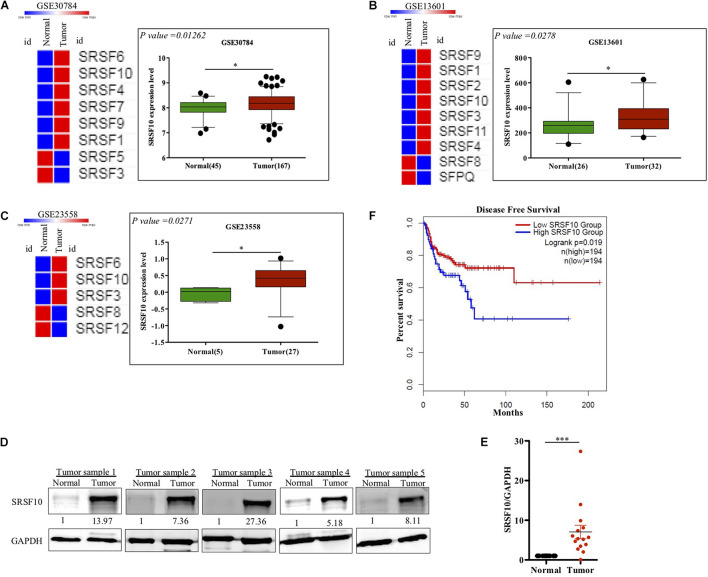
Clinical relevance of SRSF10 expression in head and neck cancer: **(A–C)** Heat map of SR splicing factor proteins in head and neck cancer profiles (analyzed using GEO database) and the expression analysis of SRSF10 in the HNC profiles downloaded from GEO database, **(A)** GSE30784, **(B)** GSE13601, **(C)** GSE23558, and **(D)** Immunoblotting showing the SRSF10 expression at the protein level in 15 head and neck cancer patient samples and the paired normal (also see the Supplementary Figure 1L), **(E)** Quantification of SRSF10 immunoblots in 15 head and neck cancer patient samples and the paired normal which is normalized to GAPDH, **(F)** Kaplan–Meier curve showing significant association (*p* = 0.019) of disease-free patient survival with SRSF10 expression in TCGA dataset. GEO, Gene Expression Omnibus; GSE, Genomic Spatial Events. Error bars show the mean values ± SD and the differences were considered statistically significant with ^∗^*P* < 0.05 and ^∗∗∗^*P* < 0.001, ns non-significant (*P* > 0.05).

SRSF10 has been reported to function as a splicing activator in a sequence-specific manner (Zhou et al., 2014a). Here we selected various independent HNC cohorts from the GEO database (Barrett et al., 2013) and analyzed them for the altered expression of all the members of SR family splicing factor. We observed deregulation of a few of SR family members; among them, the SRSF10 was commonly up-regulated in all the HNC cohorts (Figures 1A–C). Further, to support our preliminary analysis, we analyzed the HNC profiles available in the Oncomine (Rhodes et al., 2004) and observed the upregulation of SRSF10 (Supplementary Figures 1A–K) in tumor tissues as compared to normal tissue obtained from the HNC patients. Next, we validated these *in silico* analysis in the HNC tissue samples obtained from the HNC patients receiving treatment at the Bansal Hospital, Bhopal, and observed the increased SRSF10 level by immunoblotting in HNC patient’s tumor tissues as compared with the paired normal (Figure 1D and Supplementary Figure 1L). The immunoblotting included HNC tissue samples and corresponding normal tissues from 15 HNC patients. Statistical analysis showed that SRSF10 was significantly up-regulated in the HNC cancer patient samples compared with the paired normal tissues (Figure 1E). Moreover, the survival analysis using GEPIA 2.0 online platform (Gene Expression Profiling Interactive Analysis) with TCGA dataset (Figure 1F) showed that patients with low levels of SRSF10 expression had significantly longer survival than patients with higher SRSF10 expression (Figure 1F), this data includes patients with follow-up data till 200-months. We observed the similar results with Kaplan–Meier overall survival analysis using GEO dataset (GSE26549; Supplementary Figure 1M), where we used the patients with follow-up data till 10-months.

Together these observations strongly indicate that overexpression of SRSF10 was closely associated with the poor survival in HNC patients and also indicates that SRSF10 can act as an oncogenic driver in HNC. Thus, it highlights the irresistible need to identify the regulatory mechanism that underlies the increased SRSF10 level in HNC.

### EGR1 Mediated Hydroxymethylation Leads to Increased SRSF10 Expression in HNC

**FIGURE 2:**
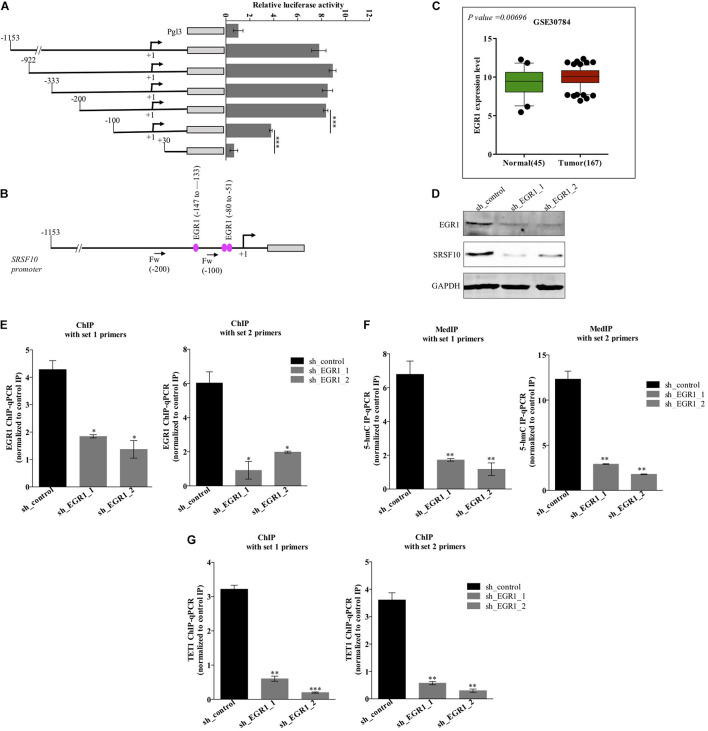
Level of EGR1 regulates the expression of SRSF10 in HNC cells BICR10: **(A)** Luciferase assay with the deletion construct of SRSF10 promoter, **(B)** schematic representation showing the binding position for EGR1, **(C)** the expression analysis of EGR1 in the HNC profiles downloaded from GEO database (GEO30784), **(D)** Immunoblot showing the protein level of EGR1, SRSF10 in sh_EGR1 transfected cells versus sh_control in BICR10 cells, GAPDH act as a loading control. **(E)** EGR1-ChIP performed in sh_EGR1 transfected BICR10 cells in comparison to sh_control using indicated primers for SRSF10 promoter region, **(F)** hMedIP experiment performed in sh_EGR1 transfected BICR10 cells in comparison to sh_control using indicated primers for SRSF10 promoter region, **(G)** TET1-ChIP performed in sh_EGR1 transfected BICR10 cells in comparison to sh_control using indicated primers for SRSF10 promoter region. Error bar represents the mean values ± SD. Differences were considered statistically significant with *P < 0.05, ^∗∗^*P* < 0.01, and ^∗∗∗^*P* < 0.001.

The function of splicing factors is regulated either by affecting their post-translational modifications or affecting the expression level, thus modulating their target gene’s splicing. Here in this study, we investigated the mechanism involved in the expression of SRSF10, the promoter region of the SRSF10 was dissected into a series of deletion fragments and constructed in a pGL3 basic vector, termed as pGL3-1153, pGL3-922, pGL3-333, pGL3-200, pGL3-100, and pGL3+30. The luciferase reporter assay was performed to detect the transcriptional activity of the fragments. In comparison to the pGL3-basic vector, the luciferase activity in all the constructs was increased, and the fragment –200 to +30 bp exhibited to decrease in the luciferase activity (Figure 2A), indicating the presence of several possible positive regulatory elements in this segment of the SRSF10 promoter, absence of which could diminish the expression of SRSF10. However, the screening of the fragment −200 to +30 bp highlights the presence of EGR1 (Figure 2B). Next, we analyzed the HNC profiles using Oncomine platform (Supplementary Figures 2B,C) as well as the HNC profiles available in the GEO database (Figure 2C) and observed the upregulation of (Early Growth Response1) EGR1 in HNC tumor tissues as compared to normal tissue. Interestingly, we also observed that the downregulation of EGR1 suppresses the expression of SRSF10 at the protein level (Figure 2D). To further understand the role of EGR1 in SRSF10 expression, we performed the ChIP using EGR1 antibody and primers specific to SRSF10 promoter fragment −200 to +30 (Supplementary Figure 2A). We observed the decrease in EGR1 enrichment at the SRSF10 promoter region in sh_EGR1 transfected cells in comparison to the sh_control HNC cell (Figure 2E). The earlier reports explain the regulatory mechanism with EGR1 mediated increase in the target genes expression as EGR1 mediated recruitment leads to the hypomethylation (hydroxymethylated) of the sites in neuronal cells (Sun et al., 2019). Therefore, we hypothesized that EGR1 leads to the increased SRSF10 expression via recruiting the TET1, which demethylate or hydroxymethylated the CpG moiety in the promoter region of SRSF10. To further understand, we performed the hMedIP experiment using the 5hmC antibody in sh_EGR1 transfected cells in comparison to sh_control cells and observed the significant downregulation of hydroxymethylation at the promoter region of SRSF10 (Figure 2F). Along the line to validate our hypothesis that EGR1 mediated recruitment of TET1 is responsible for the SRSF10 promoter demethylation (hydroxymethylation). We performed ChIP using TET1 antibody and observed the decrease in TET1 enrichment at the SRSF10 promoter region in sh_EGR1 transfected cells compared to sh_control HNC cell (Figure 2G).

**FIGURE 3:**
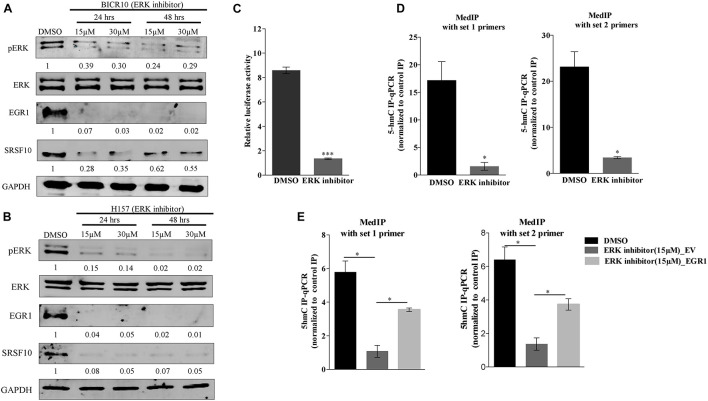
ERK/MAPK signaling pathway regulates the EGR1 and SRSF10 expression: **(A)** Immunoblot showing the protein level of pERK, ERK, EGR1, and SRSF10 in ERK inhibitor-treated cells versus DMSO in BICR10 cells, GAPDH act as a loading control, **(B)** Immunoblot showing the protein level of pERK, ERK, EGR1, and SRSF10 in ERK inhibitor-treated cells versus DMSO in H157 cells, GAPDH act as a loading control, **(C)** Luciferase assay in ERK inhibited cells transfected with the SRSF10 promoter constructs, **(D)** hMedIP experiment performed in ERK inhibitor-treated cells versus DMSO in BICR10 cells, **(E)** hMedIP experiment performed in ERK inhibitor-treated cells versus ERK inhibitor-treated cells proceed by EGR1 overexpression in comparison to DMSO control in BICR10 cells using indicated primers for SRSF10 promoter region. Error bar represents the mean values ± SD. Differences were considered statistically significant with *P < 0.05 and ^∗∗∗^*P* < 0.001.

Additionally, the upregulation of EGR1 is shown to be associated with the activation of the ERK/MAPK signaling pathway (Gregg and Fraizer, 2011). Next, to examine the role of the ERK/MAPK signaling pathway in the EGR1 to SRSF10 axis, we used a chemical compound purchased from the library of pharmacologically active compounds that target and inhibit the ERK signaling pathway. Interestingly, with the inhibition of the ERK/MAPK signaling pathway, we observed significant decrease in EGR1 expression as well as the decreased SRSF10 expression in an immunoblot analysis (Figures 3A,B), which was further confirmed by the luciferase reporter assay (Figure 3C). Further, to rule out whether the final effect of ERK/MAPK inhibition on SRSF10 expression is via EGR1 mediated demethylation at the SRSF10 promoter region. We performed the hMedIP experiment using 5hmC antibody and observed the significant decrease in hydroxymethylation in ERK inhibitor-treated sample in comparison to control (Figure 3D). Additionally, In order to validate the dependency of SRSF10 expression on EGR1, we overexpressed EGR1 in ERK inhibitor-treated BICR10 cells. The overexpression of EGR1 in ERK inhibitor treated cells rescued the expression of SRSF10 in ERK inhibitor_EGR1 cells as compared to ERK inhibitor_EV control cells (Supplementary Figure 3A). In continuation, we also examined the level of the hydroxymethylation level at SRSF10 promoter in ERK inhibitor_EGR1 samples and observed that hydroxymethylation was regained significantly by EGR1 overexpression in ERK inhibitor-treated cells in comparison to ERK inhibitor_EV cells (Figure 3E).

**FIGURE 4:**
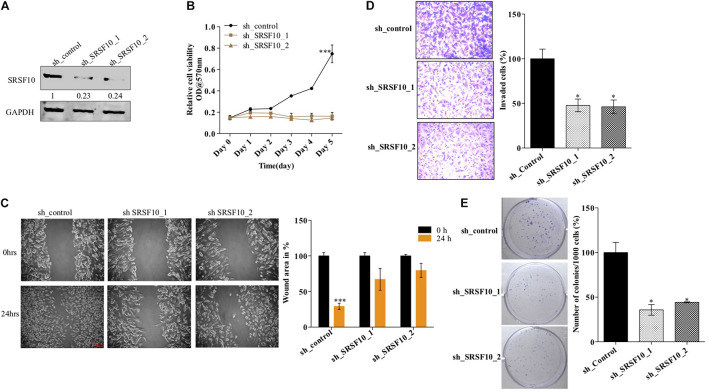
SRSF10 affects the proliferation in head and neck cancer cell BICR10: SRSF10 expression was depleted in HNC cell lines, and differential proliferation status of these cells was analyzed, **(A)** Immunoblot showing the protein level of SRSF10 in sh_SRSF10 transfected cell in comparison to sh_control cells, GAPDH acts as a loading control, **(B)** relative cell proliferation was analyzed through MTT assasy, **(C)** cell migration was analyzed through wound healing assay, **(left)** wound was observed under the microscope and the **(right)** quantification of wound width, **(D)** cell invasion assay in BICR10 cells, **(left)** representative image of invasion assay **(right)** quantification of cell count in different groups, **(E)** clonogenic assay in BICR10 cells, **(left)** representative image of clone forming cells, **(right)** quantification of cloning efficiency of BICR10 cells. Error bar represents the mean values ± SD. Differences were considered statistically significant with *P < 0.05 and ^∗∗∗^*P* < 0.001.

Furthermore, we also investigated the role of SRSF10 in tumorogenic properties in HNC cells using two independent shRNAs targeting SRSF10. Immunoblotting confirmed the sh_SRSF10 mediated SRSF10 depletion in comparison to sh_control in HNC cell lines (Figure 4A and Supplementary Figure 4A). Importantly, we observed significant growth inhibition in SRSF10 depleted cells as analyzed with the MTT assay (Figure 4B and Supplementary Figure 4B). SRSF10 depletion also remarkably reduce the wound healing capacity (Figure 4C and Supplementary Figure 4C), showing the effect of SRSF10 on cell migration and proliferation. The same is evident in the transwell cell migration assay, performed to analyze the effect of SRSF10 on single-cell motility. We analyzed that SRSF10 depletion reduced the number of invaded cells significantly in sh_SRSF10 transfected cells in comparison to sh_control cells (Figure 4D). Sequentially, to examine the role of SRSF10 in colony-forming capacity of HNC cells, we performed colony formation assay and observed decreased colony formation in SRSF10 depleted BICR10 HNC cells in comparison to control cells (Figure 4E).

These results show that SRSF10 affects the proliferation, wound healing, invasion, and colony-forming capacity of HNC cells, and thus SRSF10 overexpression in HNC might play a critical role in HNC oncogenesis. More importantly, the overexpression of SRSF10 is under control of ERK/MAPK-EGR1 axis.

### SRSF10 Upregulation Promotes HNC Progression by Favoring Cancer-Related Splicing Variants in HNC

SRSF10 was observed to play a critical role in myoblast differentiation (Wei et al., 2015) and adipocyte development (Li et al., 2014) via controlling the splicing of the critical genes, while the role of SRSF10 in HNC carcinogenesis is not yet clear. It has been shown earlier that the adipogenic defects caused by SRSF10 deficiency in mouse embryonic fibroblast, and the RNA seq data showed role of SRSF10 in mediating (pyruvate kinase M) PKM pre-mRNA splicing (Li et al., 2014). In continuation, another report explains SRSF10 mediated regulation of (BCLx apoptosis regulator) BCLx pre-mRNA splicing (Shkreta et al., 2016). These two studies caught our attention as we are aware that these two SRSF10 targets reported in the two different studies, are functionally associated with the cancer progression as the BCLx pre-mRNA splicing is related to the apoptosis of cells (Adams and Cory, 2007) and PKM pre-mRNA splicing is associated with the Warburg effect (Christofk et al., 2008).

**FIGURE 5:**
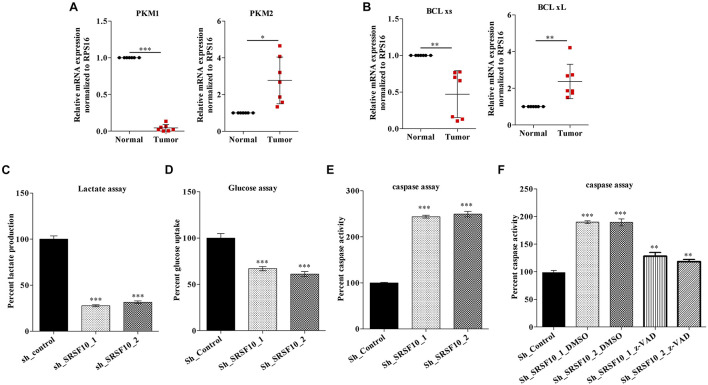
Clinical relevance of PKM and BCLx gene and effect of SRSF10 depletion on the tumorogenic potential of HNC: **(A,B)** RPS16 normalized qRT-PCR in paired normal and tumor HNC patient’s samples using the splicing primers for **(A)** PKM genes and **(B)** BCLx gene. **(C,D)** Percentage of decreased **(C)** lactate production and **(D)** glucose uptake in sh_SRSF10 transfected cells in comparison to sh_control cells, **(E)** Percentage of increase in caspase activity in sh_SRSF10 transfected cells in comparison to sh_control cells, **(F)** Percentage of caspase activity in sh_SRSF10 transfected cells in comparison to sh_control and effect of pan caspase inhibitor (z-VAD-FMK) in BICR10 cells. Three independent experiments were conducted, and the representative data are shown here with the mean values ± SD. *P* value using two-tailed student’s *t*-test, ^∗^*P* < 0.05, ^∗∗^*P* < 0.01, ^∗∗∗^*P* < 0.001, and ns = non-significant.

Next, we validated the expression of BCLx isoforms (Supplementary Figure 3B) and PKM isoforms (Supplementary Figure 3C) in the HNC tissue samples obtained from patients under treatment at the Bansal Hospital, Bhopal, and we observed the higher PKM2 and low PKM1 expression in HNC tumor tissue samples in comparison to paired normal tissue samples at the RNA level (Figure 5A). Similarly we observed higher BCLxL (cancer-specific isoform) and low BCLxs (normal isoform) expression (Figure 5B) in HNC tumor tissue samples in comparison to paired normal tissues at the RNA level. Further, as we know, the PKM2 isoform of the PKM gene is associated with the Warburg effect (Rajala et al., 2016), and an increase in the Warburg effect is indicated with the increase in lactate production and glucose uptake (Heiden et al., 2009). Thus, we hypothesized that SRSF10 regulates the PKM splicing favoring the PKM2 isoform, leading to the cancer progression via the Warburg effect. Next, we examined the effect of SRSF10 downregulation on lactate production and glucose uptake and observed the lower lactate production (Figure 5C) and decreased glucose uptake (Figure 5D) with the SRSF10 depletion as we expected. Similarly, the BCLx gene is associated with the apoptotic pathway, and here we examined the effect of SRSF10 downregulation on apoptosis via measuring the caspase activity using caspase assay and observed the increase in caspase activity with the SRSF10 depletion (Figure 5E). To rule out the promotive effect of SRSF10 downregulation in caspase assay is via BCLx pre-mRNA splicing switch from anti-apoptotic BCLxL to pro-apoptotic isoform BCLxs mediated caspase activation. We used a well-known zVAD-FMK pan-caspase inhibitor, and treated the sh_SRSF10 transfected cells, then measured the caspase activity using caspase assay (Figure 5F). We observed significant decrease in the caspase activity in pan caspase inhibitor treated sh_SRSF10 transfected cells in comparison to the sh_SRSF10_DMSO treated cells.

These observations suggest the oncogenic role of SRSF10 may partially be explained by its effect on BCLx and PKM splicing switch, which affects the Warburg effect and apoptosis and thus the growth of HNC cells.

**FIGURE 6:**
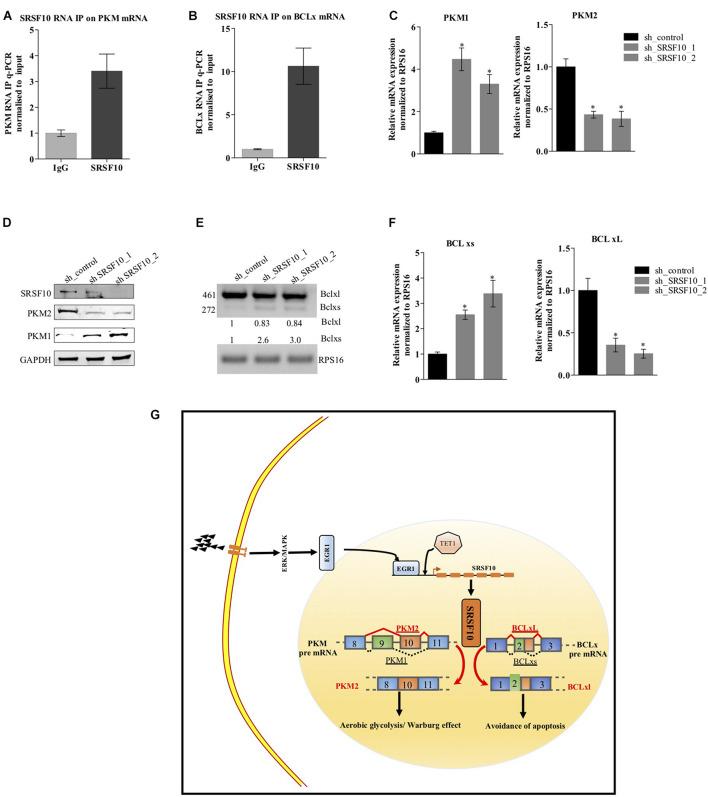
Effect of SRSF10 downregulation on splicing of PKM and BCLx gene in BICR10 cells: **(A)** qRT-PCR performed after RIP using SRSF10 antibody with constitutive primers for PKM gene, **(B)** qRT-PCR performed after RIP using SRSF10 antibody with constitutive primers for BCLx gene, **(C)** RPS16 normalized qRT-PCR in sh_SRSF10 transfected cells in comparison to sh_control using splicing primers for PKM gene, **(D)** Immunoblot showing the protein level of SRSF10, PKM1, PKM2 in sh_SRSF10 transfected cells versus sh_control in BICR10 cells, GAPDH act as a loading control, **(E)** semi-q PCR showing the two isoforms of BCLx in sh_SRSF10 transfected cells in comparison to sh_control, **(F)** RPS16 normalized qRT-PCR in sh_SRSF10 transfected cells in comparison to sh_control using splicing primers for BCLx gene, **(G)** Schematic model. Three independent experiments were conducted, and the representative data are shown here with the mean values ± SD. *P* value using two-tailed student’s *t*-test, ^∗^*P* < 0.05 and ns = non-significant.

Next, to examine the SRSF10 occupancy on BCLx and PKM gene, we performed RNA immune precipitation using the SRSF10 antibody in HNC cells, and interestingly we observed the SRSF10 enrichment at PKM RNA (Figure 6A and Supplementary Figure 5A) and BCLx RNA (Figure 6B). Further, we explored the role of SRSF10 in PKM (Figures 6C,D) as well as BCLx (Figures 6E,F) pre-mRNA splicing. We depleted the SRSF10 in HNC cells, where we observed the switch in splicing from cancer-specific isoform to normal isoform in SRSF10 down-regulated cells in comparison to the control cells at the mRNA and protein level (Figures 6C–F and Supplementary Figures 5B–E). We also observed the splicing switch from cancer-specific isoform to normal isoform in EGR1 depleted cells (Supplementary Figures 5F,G). These results collectively support the hypothesis that EGR1 plays an important role in the oncogenic effect of SRSF10 in HNC by regulating the splicing of its target genes which are known to be associated with cancer progression.

Together, these results suggest the ERK1/2-EGR1-SRSF10 axis in the generation of PKM2 and BCL-xL, cancer-specific splice isoforms as shown in the schematic diagram (Figure 6G).

## Discussion

Alternative splicing is a highly regulated process that contributes to the proteome diversity in eukaryotic organisms. The process of AS is found to be deregulated in cancer which, in turn, favors the tumor progression (Ladomery, 2013). Expression of cancer-specific isoforms of various genes in cancer cells is majorly due to epigenetic modifications at the gene locus (Singh et al., 2017) as well as due to the deregulation of splicing factors which include the SR protein family and HnRNP family members (Gupta et al., 2020). In fact, a large number of splicing factors have been reported to be deregulated in multiple cancer types, and have been found to be responsible for aberrant AS. Our study is focused on SRSF10, which is a new member of an expanded family of SR splicing factors and acts as a sequence-dependent splicing regulator (Zhou et al., 2014a). Since the characterization of the SRSF10 splicing factor in 2001 (Cowper et al., 2001), many researchers have explained the role of SRSF10 in several model systems, where they showed its importance in the developmental processes of different model systems like adipocyte development (Li et al., 2014) and myoblast development (Wei et al., 2015). While SRSF10 has been reported in different model systems including colon cancer (Zhou et al., 2014b), cervical cancer (Liu et al., 2018) for its role in the regulation of AS, its deregulation in HNC has remained to be elucidated, which is the sixth most common cancer worldwide (Parkin et al., 2005). Here in this study, we identified SRSF10 overexpression in HNC for the first time, and SRSF10 expression level is inversely related to the patient’s survival as the percent survival of the SRSF10-high group was significantly lower than the SRSF10-low group.

Our study also demonstrated that the downregulation of the SRF10 reduces the cells proliferation, migration, invasive property as well as the colony-forming ability of HNC cells, these *in vitro* analysis further support that SRSF10 plays an essential role in HNC cell growth. Our study provide a strong evidence that SRSF10 directly regulates the AS of BCLx and PKM pre-mRNA as shown by RNA immune-precipitation. Further, to analyze the role of SRSF10 in PKM and BCLx pre-mRNA splicing, we examined the PKM and BCLx pre-mRNA-splicing pattern in SRSF10 depleted HNC cells and observed the splicing switch of PKM and BCLx gene from cancer-specific (PKM2 and BCLxL) isoform to normal isoform (PKM1 and BCLxs).

We then concluded that the tumorigenic effect of SRSF10 is mediated via modulating the splicing of the genes like PKM and BCLx. Interestingly, the PKM2 isoform of the PKM gene is associated with the Warburg effect or aerobic glycolysis (Christofk et al., 2008). Warburg effect is a hallmark of cancer which is characterized by increased glucose uptake and lactate production (DeBerardinis et al., 2008) where the PKM gene regulates the key step of glycolysis, and thus the splicing switch from PKM1 to cancer-specific PKM2 isoforms plays a key role in the Warburg effect (Christofk et al., 2008; Dayton et al., 2016). Similarly, the BCLx gene is associated with apoptosis where the small isoform of BCLx that is BCLxs isoform is reported to promote the apoptosis and known as pro-apoptotic, and BCLxL isoform is the long isoform of the BCLx gene and is involved in the anti-apoptotic pathway (Boise et al., 1993; Adams and Cory, 2007). Together, these results suggest that SRSF10 promotes the cancer-specific isoforms of genes like PKM and BCLx thus play a crucial role in HNC.

To date, the role of SRSF10 in different model systems has been shown but what leads to an increase in the expression of SRSF10 in tumor samples in comparison to normal is not yet clear.

In this study, we dissected the mechanism responsible for the increased expression level of SRSF10. To study the cause of increased SRSF10 expression, we performed luciferase assay with deletion constructs of the SRSF10 promoter region, which lead to the EGR1 gene, a transcription factor. Interestingly, EGR1 is shown to function as an oncogene in prostate cancer (Virolle et al., 2003), and EGR1 mediated expression of its target genes involves EGR1mediated recruitment of TET1 at the EGR1 binding site which further promote the demethylation (hydroxymethylation), elevating the expression of the EGR1 target gene in neuronal cells (Sun et al., 2019). Interestingly, in HNC cells we observed similar regulatory mechanism of EGR1 binding at the SRSF10 promoter region and EGR1 mediated recruitment of the TET1 at the EGR1 binding site leading to demethylation or hydroxymethylation of the EGR1 site, thus increasing the expression of SRSF10. Though, our results explain the role of EGR1 as a regulator of SRSF10 expression via TET1 recruitment, but further studies will be needed to understand if the TET1 acts as a docking site for other co-factors to co-operate with EGR1.

Next, with the literature support we analyzed the role of ERK/MAPK pathway in the regulation of EGR1 expression level. We observed the decrease in EGR1 and SRSF10 expression at the protein level with the inhibition of ERK1/2 phosphorylation. The effect of ERK/MAPK inhibition on SRSF10 expression was also confirmed with luciferase activity, and the expression level of SRSF10 was rescued with the overexpression of EGR1 in ERK inhibitor-treated cells. Together, these results support the ERK/MAPK-EGR1-SRSF10 axis is crucial for HNC progression and provide an alternative strategy for drug targets.

Collectively, these results suggest the ERK 1/2-EGR1-SRSF10 axis, which could explain the SRSF10 overexpression and its regulation in line with the ERK1/2 pathway via the EGR1 transcription factor. To confirm the axis of the ERK pathway to SRSF10 via EGR1, we performed the rescue experiment with the EGR1 overexpression construct where we overexpressed the EGR1 in ERK1/2 inhibitor-treated cells and observed the increase in SRSF10 protein expression level, which indicated that the ERK1/2-EGR1-SRSF10 axis plays a role in modulating the SRSF10 targeted splicing.

## Data Availability Statement

Publicly available datasets were analyzed in this study. This data can be found here: https://www.ncbi.nlm.nih.gov/geo/, GSE26549, GSE30784, GSE13601, and GSE23558.

## Ethics Statement

The studies involving human participants were reviewed and approved by Institute Ethics Committee, Indian Institute of Science Education and Research Bhopal. The patients/participants provided their written informed consent to participate in this study.

## Author Contributions

SS and SY designed the experiments, wrote the manuscript, and analyzed the data. SY performed the western blotting, luciferase assay, ChIP, MedIP, RIP, qPCR, MTT assay, wound healing, Lactate assay, Glucose assay, Caspase assay, invasion assay, colony formation assay, bioinformatics analysis, and wrote the manuscript. DP performed the Kaplan–Meier survival curve analysis. AS provided the clinical samples and histopathological information. SS, NK, and SG contributed to conceptualization, formal analysis, visualization and wrote, review, and edited the manuscript. All authors contributed to the article and approved the submitted version.

## Conflict of Interest

The authors declare that the research was conducted in the absence of any commercial or financial relationships that could be construed as a potential conflict of interest.

## Publisher’s Note

All claims expressed in this article are solely those of the authors and do not necessarily represent those of their affiliated organizations, or those of the publisher, the editors and the reviewers. Any product that may be evaluated in this article, or claim that may be made by its manufacturer, is not guaranteed or endorsed by the publisher.

## References

[B1] Adams J. M. Cory S. (2007). The Bcl-2 apoptotic switch in cancer development and therapy. *Oncogene* 26 1324–1337. 10.1038/sj.onc.1210220 17322918 PMC2930981

[B2] Gupta A. Sandhya Y. Archana J. M. Atul S. Rajendra K. P. Sanjeev S. (2020). The HNRNPA2B1–MST1R–Akt axis contributes to epithelial-to-mesenchymal transition in head and neck cancer. *Laboratory Investigation* 100 1589–1601. 10.1038/s41374-020-0466-8 32669614

[B3] Barrett T. Wilhite S. E. Ledoux P. Evangelista C. Kim I. F. Tomashevsky M. (2013). NCBI GEO: Archive for functional genomics data sets - Update. *Nucleic Acids Research* 41 D991–D995. 10.1093/nar/gks1193 23193258 PMC3531084

[B4] Blaustein M. Pelisch F. Srebrow A. (2007). Signals, pathways and splicing regulation. *International Journal of Biochemistry and Cell Biology* 39 2031–2048. 10.1016/j.biocel.2007.04.004 17507279

[B5] Boise L. H. González-García M. Postema C. E. Ding L. Lindsten T. Turka L. A. (1993). bcl-x, a bcl-2-related gene that functions as a dominant regulator of apoptotic cell death. *Cell* 74 597–608. 10.1016/0092-8674(93)90508-N 8358789

[B6] Boukakis G. Patrinou-Georgoula M. Lekarakou M. Valavanis C. Guialis A. (2010). Deregulated expression of hnRNP A/B proteins in human non-small cell lung cancer: Parallel assessment of protein and mRNA levels in paired tumour/non-tumour tissues. *BMC Cancer* 10:434. 10.1186/1471-2407-10-434 20716340 PMC2933625

[B7] Christofk H. R. Vander Heiden M. G. Harris M. H. Ramanathan A. Gerszten R. E. Wei R. (2008). The M2 splice isoform of pyruvate kinase is important for cancer metabolism and tumour growth. *Nature* 452 230–233. 10.1038/nature06734 18337823

[B8] Cowper A. E. Cáceres J. F. Mayeda A. Screaton G. R. (2001). Serine-Arginine (SR) Protein-like Factors That Antagonize Authentic SR Proteins and Regulate Alternative Splicing. *Journal of Biological Chemistry* 276 48908–48914. 10.1074/jbc.M103967200 11684676

[B9] Dayton T. L. Jacks T. vander Heiden M. G. (2016). PKM 2, cancer metabolism, and the road ahead. *EMBO reports* 17 1721–1730. 10.15252/embr.201643300 27856534 PMC5283597

[B10] DeBerardinis R. J. Lum J. J. Hatzivassiliou G. Thompson C. B. (2008). The Biology of Cancer: Metabolic Reprogramming Fuels Cell Growth and Proliferation. *Cell Metabolism* 7 11–20. 10.1016/j.cmet.2007.10.002 18177721

[B11] Dvinge H. Kim E. Abdel-Wahab O. Bradley R. K. (2016). RNA splicing factors as oncoproteins and tumour suppressors. *Nature Reviews Cancer* 16 413–430. 10.1038/nrc.2016.51 27282250 PMC5094465

[B12] Liu F. Dai M. Xu Q. Zhu X. Zhou Y. Jiang S. (2018). SRSF10-mediated IL1RAP alternative splicing regulates cervical cancer oncogenesis via mIL1RAP-NF-κB-CD47 axis. *Oncogene* 37 2394–2409. 10.1038/s41388-017-0119-6 29429992 PMC5931977

[B13] Garg P. Karjodkar F. (2012). Catch them before it becomes too late”-oral cancer detection. Report of two cases and review of diagnostic AIDS in cancer detection. *International Journal of Preventive Medicine* 3 737–741. 23112903 PMC3483004

[B14] Gregg J. Fraizer G. (2011). Transcriptional Regulation of EGR1 by EGF and the ERK Signaling Pathway in Prostate Cancer Cells. *Genes and Cancer* 2 900–909. 10.1177/1947601911431885 22593802 PMC3352154

[B15] Heiden M. G. V. Cantley L. C. Thompson C. B. (2009). Understanding the warburg effect: The metabolic requirements of cell proliferation. *Science* 324 1029–1033. 10.1126/science.1160809 19460998 PMC2849637

[B16] Li H. Yuanming Cheng Wenwu Wu Yuguo Liu Ning Wei Xiaoyan Feng, et al. (2014). SRSF10 Regulates Alternative Splicing and Is Required for Adipocyte Differentiation. *Molecular and Cellular Biology* 34 2198–2207. 10.1128/mcb.01674-13 24710272 PMC4054296

[B17] Kumar D. (2017). Regulation of glycolysis in head and neck squamous cell carcinoma. *Postdoc Journal* 5 14–28. 10.14304/surya.jpr.v5n1.4 28191478 PMC5300767

[B18] Ladomery M. (2013). Aberrant alternative splicing is another hallmark of cancer. *International Journal of Cell Biology* 2013 463786. 10.1155/2013/463786 PMC3786539 24101931

[B19] Nigro L. C Denaro N. Merlotti A. Merlano M. (2017). Head and neck cancer: Improving outcomes with a multidisciplinary approach. *Cancer Management and Research* 18 363–371. 10.2147/CMAR.S115761 28860859 PMC5571817

[B20] Shkreta L. Johanne T. Mathieu D. Manley J. L Benoit C. (2016). SRSF10 Connects DNA Damage to the Alternative Splicing of Transcripts Encoding Apoptosis, Cell-Cycle Control, and DNA Repair Factors. *Cell Reports* 17 1990–2003. 10.1016/j.celrep.2016.10.071 27851963 PMC5483951

[B21] Parkin D. M. Bray F. Ferlay J. Pisani P. (2005). Global Cancer Statistics, 2002. *CA: A Cancer Journal for Clinicians* 55 74–108. 10.3322/canjclin.55.2.74 15761078

[B22] Rajala R. V. S Ammaji R. Christopher K. Yuhong W. Anderson R. E (2016). The warburg effect mediator pyruvate kinase M2 expression and regulation in the retina. *Scientific Reports* 6 37727. 10.1038/srep37727 27883057 PMC5121888

[B23] Rhodes D. R Jianjun Y. Shanker K. Nandan D. Radhika V. Debashis G. (2004). ONCOMINE: A Cancer Microarray Database and Integrated Data-Mining Platform. *Neoplasia* 6 1–6. 10.1016/s1476-5586(04)80047-2 15068665 PMC1635162

[B24] Yadav S. Bhagat S. D Amit G. Atul S. Aasheesh S. Sanjeev S. (2019). Dietary-phytochemical mediated reversion of cancer-specific splicing inhibits Warburg effect in head and neck cancer. *BMC Cancer* 19:1031. 10.1186/s12885-019-6257-1 31675998 PMC6823945

[B25] Shin C. Feng Y. Manley J. L. (2004). Dephosphorylated SRp38 acts as a splicing repressor in response to heat shock. *Nature* 427 553–558. 10.1038/nature02288 14765198

[B26] Silipo M. Gautrey H. Tyson-Capper A. (2015). Deregulation of splicing factors and breast cancer development. *Journal of Molecular Cell Biology* 7 388–401. 10.1093/jmcb/mjv027 25948865

[B27] Singh S. Sathiya P. N. Kajal B. Amit G. Neha A. Sandhya Y. (2017). Intragenic DNA methylation and BORIS-mediated cancer-specific splicing contribute to the Warburg effect. *Proceedings of the National Academy of Sciences of the United States of America* 114 11440–11445. 10.1073/pnas.1708447114 29073069 PMC5664520

[B28] Srebrow A. Kornblihtt A. R. (2006). The connection between splicing and cancer. *Journal of Cell Science* 119 2635–2641. 10.1242/jcs.03053 16787944

[B29] Virolle T. Anja K. -H. Veronique B. De Gregorio G. Adamson E. D Dan M. (2003). Egr1 promotes growth and survival of prostate cancer cells: Identification of novel Egr1 target genes. *Journal of Biological Chemistry* 278 11802–11810. 10.1074/jbc.M210279200 12556466

[B30] Wei N. Cheng Y. Wang Z. Liu Y. Luo C. Liu L. (2015). SRSF10 Plays a Role in Myoblast Differentiation and Glucose Production via Regulation of Alternative Splicing. *Cell Reports* 13 2015. 10.1016/j.celrep.2015.10.038 26586428

[B31] Zhou X. Wenwu W. Huang L. Yuanming C. Ning W. Jie Z. (2014a). Transcriptome analysis of alternative splicing events regulated by SRSF10 reveals position-dependent splicing modulation. *Nucleic Acids Research* 42 4019–4030. 10.1093/nar/gkt1387 24442672 PMC3973337

[B32] Zhou X. Xuebing L. Yuanming C. Wenwu W. Zhiqin X. Qiulei X. (2014b). BCLAF1 and its splicing regulator SRSF10 regulate the tumorigenic potential of colon cancer cells. *Nature Communications* 5 4581. 10.1038/ncomms5581 25091051

[B33] Sun Z. Xiguang Xu Jianlin He Alexander Murray Ming-an Sun Xiaoran Wei (2019). EGR1 recruits TET1 to shape the brain methylome during development and upon neuronal activity. *Nature Communications* 10 1–12. 10.1038/s41467-019-11905-3 31467272 PMC6715719

