# Supplementary material for: ERK1/2-EGR1-SRSF10 Axis Mediated Alternative Splicing Plays a Critical Role in Head and Neck Cancer
Source: Front Cell Dev Biol. 2021 Sep 20;9:713661. doi: 10.3389/fcell.2021.713661 (PMC8489685; doi:10.3389/fcell.2021.713661)
Supplement: Supplementary file 1 [file Data_Sheet_1.pdf]

Supplementary Tables

Table S1: List of primer sequences utilized for qPCR and clonning

S.No.	Primer	Sequence
1	PKM 11 Fw	CCATCATTGCTGTGACCCGGAAT
2	PKM 11 Rev	CATTCATGGCAAAGTTCACCCGGA
3	PKM 8-9 Fw	ATGCAGCACCTGATAGCTCGTGA
4	PKM 9 Rev	GTTCCACCGCAAGCTGTTTGAAGA
5	PKM 10-11 Fw	TCACCAAGTCTGGCAGGTCTG
6	RPS16 Fw	AAACGCGGCAATGGTCTCATCAAG
7	RPS16 Rev	TGGAGATGGACTGACGGATAGCAT
8	Bcl xs exon 2-3Fw	TATCAGAGCTTTGAACAGGATAC
9	Bcl xs exon 3Rev	AAGAGTGAGCCCAGCA
10	Bcl xL exon 2Fw	TCCTTCGGCGGGGCAC
11	Bcl xL exon 2Rev	ACCCAGCCGCCGTTCT
12	Bclx const. Fw	TTGTGGAACTCTATGGGAACAA
12	Bclx const. Rev	TCATTTCCGACTGAAGAGTGAG
13	Bclx semiq Rev	GAGGCAGGCGACGAGTTTGAA
13	Bclx semiq Rev	TGGGAGGGTAGAGTGGATGGT
14	EGR1 OE Fw	CCGGAATTCATGGCCGCGGCCAAGGCCGA
15	EGR1 OE Rev	CCGCTCGAGTTAGCAAATTTCAATTGTCC
16	SRSF10 promoter -1153 Fw	CGGCTAGCACGGAGTCTCACCCTGTC
17	SRSF10 promoter -922 Fw	CGGCTAGCCCAGCCGAAGTTTCCTCTATTT
18	SRSF10 promoter -333 Fw	CGGCTAGCGATGAAGTGGAGTGTAAGGTAGAA
19	SRSF10 promoter -200 Fw	CGGCTAGCAGAAGCCAACGAATGACACC
20	SRSF10 promoter -100 Fw	CGGCTAGCACTTTGCGCGGAGGTA
21	SRSF10 promoter +30 Fw	CGGCTAGCTTGCTGAGCCCGTTAGTG
22	SRSF10 promoter +300 Rev	CCCAAGCTTGACCCGCCATCTTCACTC
23	SRSF10 promoter Fw set1	AGAAGCCAACGAATGACACC
24	SRSF10 promoter Rev set1	CTACCTCCGCGCAAAGTC
25	SRSF10 promoter Fw set2	GACTTTGCGCGGAGGTA
26	SRSF10 promoter Rev sert2	CTCAGACACACACAGCTAGAG

Table S2: Clinical characteristics of patients.

S.No.	Patient	Histopathology
1	Patient 1	Carcinoma of right buccal mucosa
2	Patient 2	Carcinoma of left buccal mucosa
3	Patient 3	Carcinoma of left buccal mucosa
4	Patient 4	Carcinoma of left RMT(retromolar trigone) with involvement of ITF (infratemporal fossa)
5	Patient 5	Carcinoma of left lower gingivobuccal sulcus (GBS)
6	Patient 6	Carcinoma of right lower gingivobuccal sulcus
7	Patient 7	Carcinoma of right lateral border of tongue
8	Patient 8	Carcinoma of right lower gingivobuccal sulcus
9	Patient 9	Carcinoma of floor of mouth, involves tongue
10	Patient 10	Carcinoma of tongue
11	Patient 11	Carcinoma of right buccal mucosa
12	Patient 12	Carcinoma of left buccal mucosa
13	Patient 13	Carcinoma of left upper alveolus
14	Patient 14	Carcinoma of right buccal mucosa with carcinoma in left upper GBS
15	Patient 15	Carcinoma of lower alveolus

Legends to Supplementary figures

Supplementary figure 1: Clinical relevance of SRSF10 expression in head and neck cancer: SRSF10 expression analysis in different HNC profiles using oncomine platform, **A-D)** Pyeon multi cancer, **E)** Toruner Head-Neck, **F)** FriersonHF Salivary-gland**, G)** Talbot Lung, **H)** Ye Head-Neck, **I)** Estilo Head-Neck, **J)** Ginos Head-Neck, **K)** Sengupta Head-Neck, **L)** Immunoblot showing the SRSF10 expression at the protein level in head and neck cancer patient samples and the paired normal, **M)** The 10 month survival data of HNC patient obtained from gene expression omnibus (GSE26549) and Kaplan-Meier survival curve analysis between a group of low SRSF10 and high SRSF10 expressing patients

Supplementary Figure 2: EGR1 expression in head and neck cancer profiles: (A) schematic representation showing the binding position for EGR1 indicating the primers for ChIP and hMeDIP in SRSF10 promoter region, **(B-C)** EGR1 expression analysis using oncomine platform **b)** Ginos Head-Neck, **c)** FriesrsonHF Head-Neck.

Supplementary Figure 3: EGR1 mediated effect on SRSF10 expression level and schematic representation of Splicing pattern for SRSF10 targets: (A) Immunoblot showing the protein level of Flag, pERK, SRSF10 in ERK inhibitor cells versus ERK inhibitor-treated cells proceed by EGR1 overexpression in comparison to DMSO control in BICR10 cells, GAPDH act as a loading control, **(B)** Schematic representation of BCLx pre-mRNA-splicing pattern, **(C)** Schematic representation of PKM pre-mRNA-splicing pattern.

Supplementary figure 4: SRSF10 affects the proliferation in head and neck cancer cell H157: SRSF10 expression was depleted in two types of HNC cell lines, and differential proliferation status of these cells was analyzed, **(A)** Immunoblot showing the protein level of SRSF10 in sh_SRSF10 transfected cell in comparison to sh_control cells, GAPDH acts as a loading control, **(B)** relative cell proliferation was analyzed through MTT assasy, **(C)** cell migration was analyzed through wound healing assay, **(left)** wound was observed under the microscope and the **(right)** quantification of wound width. Error bar represents the mean values ± SD. Differences were considered statistically significant with ***P<0.001.

Supplementary figure 5: Effect of SRSF10 downregulation on splicing of PKM and BCLx gene in H157 cells: (A) qRT-PCR performed after RIP with SRSF10 antibody, **(B)** RPS16 normalized qRT-PCR in sh_SRSF10 transfected cells in comparison to sh_control using splicing primers for PKM gene, **(C)** Immunoblot showing the protein level of SRSF10, PKM1, PKM2 in sh_SRSF10 transfected cells versus sh_control in BICR10 cells, GAPDH act as a loading control. **(D)** semi-q PCR showing the two isoforms of BCLx in sh_SRSF10 transfected cells in comparison to sh_control, **(E)** RPS16 normalized qRT-PCR in sh_SRSF10 transfected cells in comparison to sh_control using splicing primers, **(F-G)** RPS16 normalized qRT-PCR in sh_EGR1 transfected BICR10 cells in comparison to sh_control using indicated primers for **(F)** PKM gene and **(G)** BCLx gene. Three independent experiments were conducted, and the representative data are shown here with the mean values ± SD. *P* value using two-tailed student's t-test, * P < 0.05, ** P < 0.01.
