# Supplementary figures and images for: ERK1/2-EGR1-SRSF10 Axis Mediated Alternative Splicing Plays a Critical Role in Head and Neck Cancer
Source: Front Cell Dev Biol. 2021 Sep 20;9:713661. doi: 10.3389/fcell.2021.713661 (PMC8489685; doi:10.3389/fcell.2021.713661)

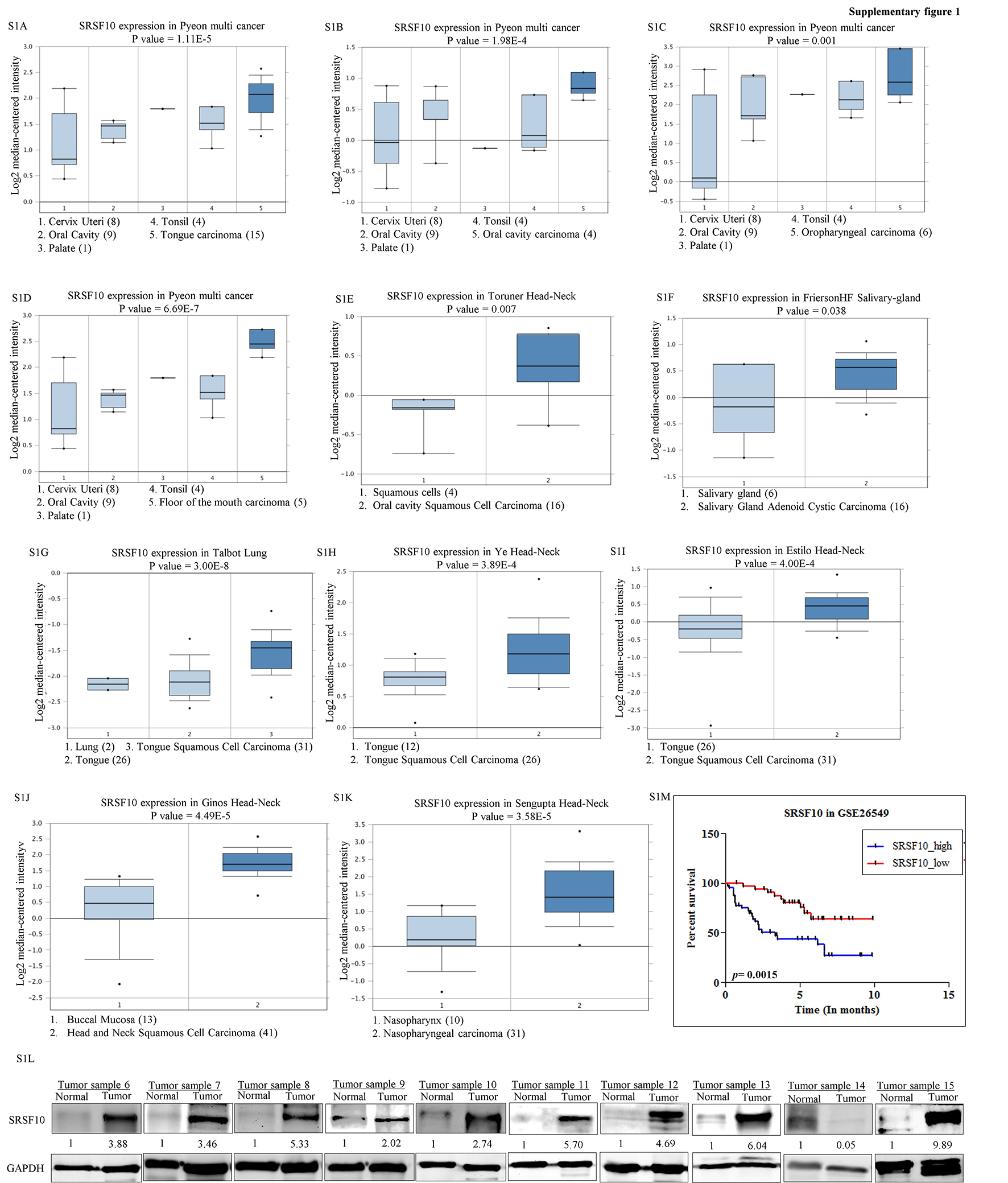

Supplement: Supplementary file 2 [file Image_1.JPEG]

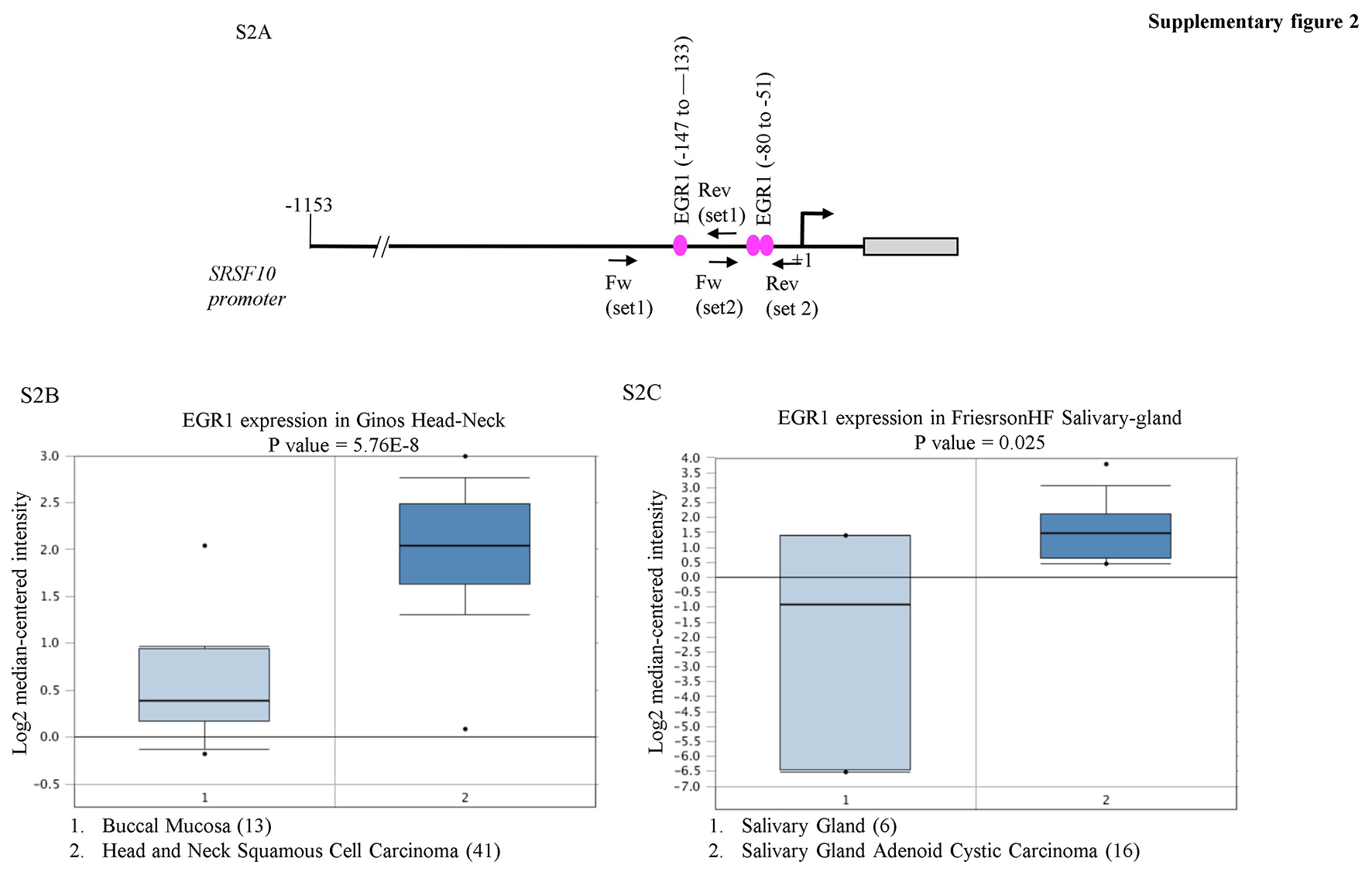

Supplement: Supplementary file 3 [file Image_2.JPEG]

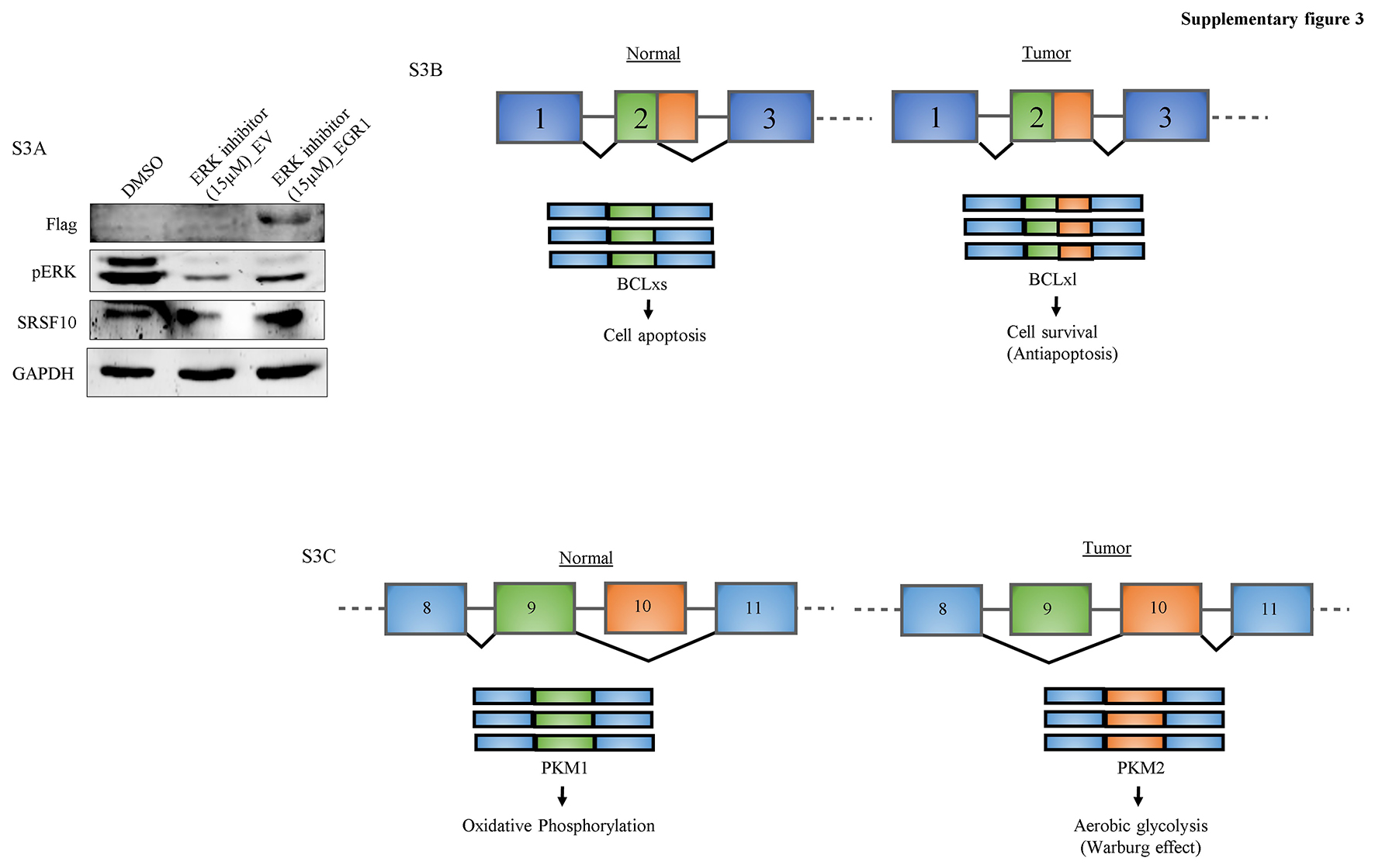

Supplement: Supplementary file 4 [file Image_3.JPEG]

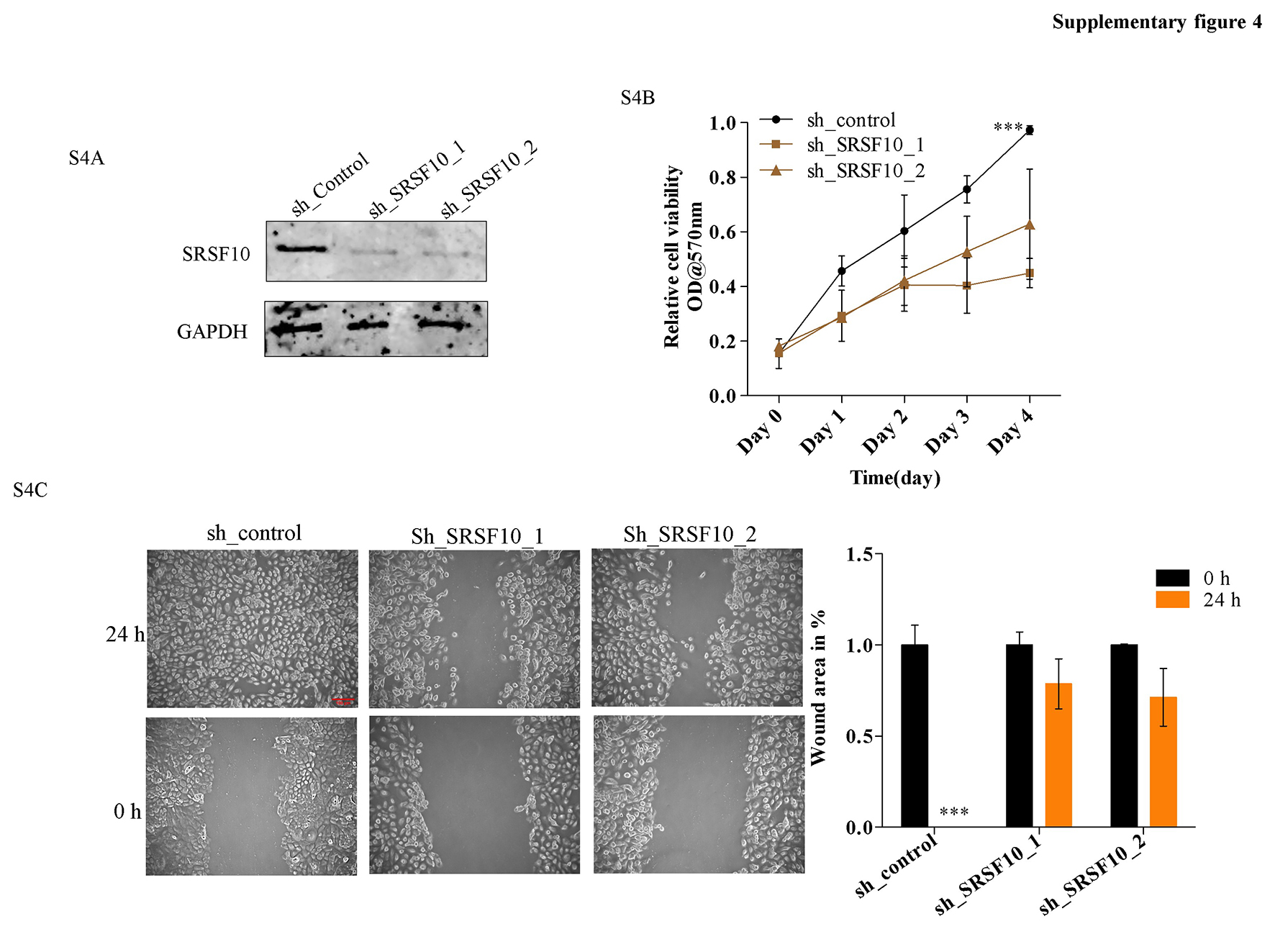

Supplement: Supplementary file 5 [file Image_4.JPEG]

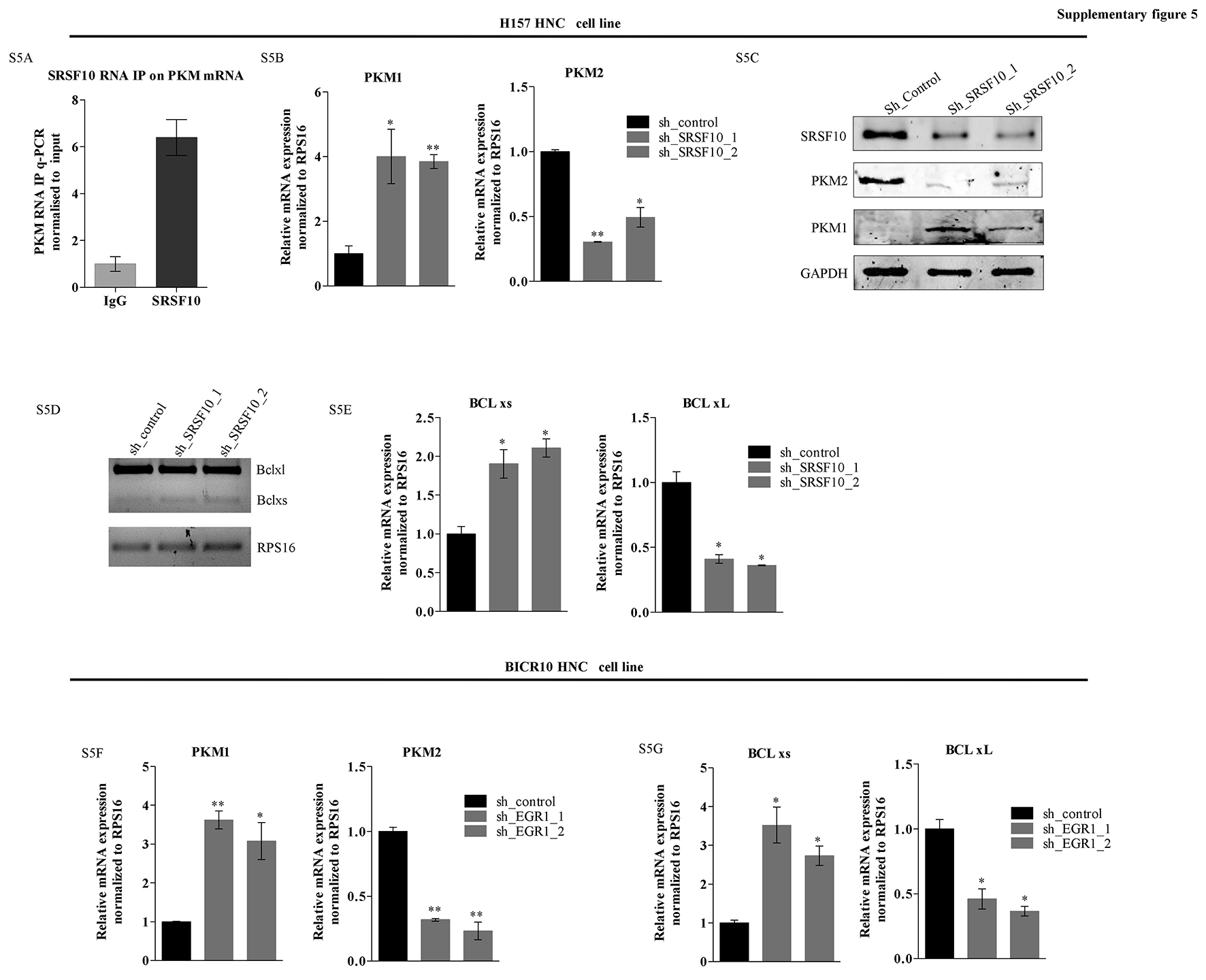

Supplement: Supplementary file 6 [file Image_5.JPEG]
